# Associations between Brain Structural Damage and Core Muscle Loss in Patients with Parkinson’s Disease

**DOI:** 10.3390/jcm9010239

**Published:** 2020-01-16

**Authors:** Ying-Nong Wu, Meng-Hsiang Chen, Pi-Ling Chiang, Cheng-Hsien Lu, Hsiu-Ling Chen, Chiun-Chieh Yu, Yueh-Sheng Chen, Yung-Yee Chang, Wei-Che Lin

**Affiliations:** 1 Department of Diagnostic Radiology, Kaohsiung Chang Gung Memorial Hospital and Chang Gung University College of Medicine, 123 Ta-Pei Road, Niao-Sung, Kaohsiung 83305, Taiwan; echo7221@gmail.com (Y.-N.W.); sperfect@msn.com (M.-H.C.); lovage@cgmh.org.tw (P.-L.C.); suring.tw@gmail.com (H.-L.C.); yuchiunchieh@gmail.com (C.-C.Y.); ggggggg4545@gmail.com (Y.-S.C.); 2 Department of Neurology, Kaohsiung Chang Gung Memorial Hospital and Chang Gung University College of Medicine, 123 Ta-Pei Road, Niao-Sung, Kaohsiung 83305, Taiwan; chlu99@adm.cgmh.org.tw (C.-H.L.); changyy7@gmail.com (Y.-Y.C.)

**Keywords:** gray matter, muscle atrophy, neurodegenerative disorder, Parkinson’s disease, voxel-based morphometry

## Abstract

Background: Parkinson’s disease (PD) is a common neurodegenerative disease associated with progressive gray matter atrophy. In addition to motor function disorder, frailty and decreased muscle mass potentially contribute to increased morbidity risk. Objective: This study aimed to investigate the associations between lean muscle loss and gray matter volume (GMV) in PD patients. Methods: Thirty patients with PD and fifteen healthy controls underwent brain and bilateral thigh MRIs. The IDEAL sequence was employed, measuring the regions of interest (ROI) of fat percentage at the 50% point of femur length. Voxel-base morphometry (VBM) was used to assess regional gray matter volume differences between groups. Further correlation analysis was performed to evaluate the changes between gray matter volume and fatty percentage of the bilateral thigh after adjusting for age and gender. Multiple linear regression analysis was applied to evaluate the risk factor of core muscle loss in PD patients. Results: Compared with controls, patients with PD had significantly higher thigh fat percentage and smaller gray matter volume of several brain locations of the default mode network (DMN), specifically the left superior temporal gyrus, right uncus, and left inferior temporal gyrus, revealing association with higher thigh fat percentage. Further multiple linear regression analysis indicated that higher thigh fat percentage is associated with gender (female), increased disease duration, and smaller gray matter volume of the left superior temporal gyrus and right uncus in PD patients. Conclusions: Patients with PD experience core muscle loss in the thigh, associated with default mode network (DMN) degeneration, longer disease duration, and female gender. Identification of risk factors associated with lean muscle mass loss may assist in early prevention of comorbidities such as sarcopenia.

## 1. Introduction

Parkinson’s disease (PD) is estimated to affect approximately 1% of the population over 60 years of age [1]. The three cardinal motor features in PD are rest tremor, rigidity, and bradykinesia. In addition to movement manifestations, several non-motor features, such as autonomic dysfunction, cognitive and psychiatric changes, dementia, sensory symptoms, and sleep disturbances are also commonly presented during disease progression [2]. Recently, physical frailty related to reduced physical activity has been identified as a predictor of decreased lifespan [3]. In PD, decreased physical activity and subsequent weakness occurs with disease progression [4]. The decreased muscle force production and general muscle weakness is associated with a deficit in the central activation of muscles [5]. Generalized loss of skeletal muscle mass and strength lead to sarcopenia, increasing the risk of adverse outcomes [6]. Several mechanisms involving advanced age, immobility, neurodegeneration, malnutrition, endocrine disorders, and cachexia in PD patients may be associated with the development of sarcopenia [7]. Therefore, it seems that neurologic deficits at various levels, from the brain to the neuromuscular junctions, could result in this adverse condition. To date, however, the relationship between muscle loss and neurological deficit has yet to be conclusively demonstrated.

PD is primarily associated with the loss of nigrostriatal dopaminergic neurons, which leads to striatal dopamine deafferentation, and causes the classic extrapyramidal motor impairment [2]. Furthermore, brain atrophy related to decreased motor function is a consequence of muscle disuse [8]. Other brain area deficits include the executive attention network and default mode network (DMN) deterioration [9,10,11]. The executive attention network dysfunction impairs both the processing of motivational and gait function, leading to further decrease of physical activity [9]. Meanwhile, default mode network dysfunction has been observed in many neurodegenerative disorders, including Parkinson’s disease [10]. A recent study reported that movement disorders, including akinesia and rigidity, are related to decreased activity in the default mode network in PD patients [11]. In addition, studies of aging have revealed that frail subjects are associated with elevated gray matter volume atrophy compared with non-frail subjects [12,13]. However, clarification of the pathophysiology within the brain and the association with lean muscle loss in PD patients require further investigation.

Numerous imaging techniques may provide effective insight in body composition analysis. Magnetic resonance imaging (MRI) techniques, such as chemical shift imaging techniques, effectively separate fat and water proton signals according to their different precession velocities, and have been widely applied for the classification of fat distribution into subcutaneous; visceral; and, more recently, intermuscular fat [14]. The iterative decomposition of water and fat with echo asymmetry and least-squares estimation (IDEAL) technique, may be particularly useful for evaluations of intramuscular component alterations [15], and demonstrates potential as a promising new biomarker for early sarcopenia detection in PD patients.

Frailty and decreased muscle mass have been observed in patients with PD, causing morbidity and mortality [3]. It is known that many factors may influence muscle integrity [7], whereas the central nervous system plays a crucial role for maintenance of muscle integrity. We hypothesized that certain structural changes in the brain may be correlated with sarcopenia in PD patients. In the present study, we applied the IDEAL technique to determine whether PD patients present with higher percentages of fat content in core and extremity muscles, and lower muscle mass than normal controls; furthermore, we deemed a comprehensive assessment of the structural changes within the entire brain necessary to more effectively evaluate the relationship between core muscle loss and specific brain structural changes.

## 2. Materials and Methods

To investigate the associations between lean muscle loss and gray matter volume (GMV) in PD patients, 30 PD patients and 15 normal controls were enrolled in our study. All studies were reviewed and approved by the Institutional Review Board of Chang Gung Memorial Hospital (Approval No. 103-6906A3) and conducted following the Declaration of Helsinki. All participants provided written informed consent following a detailed explanation of the prospective studies.

### 2.1. Study Patients

This prospective study enrolled 30 patients (10 men and 20 women; mean age: 64.90 ± 10.08 years; Hoehn and Yahr 1–2; mean disease duration: 1.13 ± 1.36 years) with idiopathic PD as diagnosed by an experienced neurology specialist in the Chang Gung Memorial Hospital Neurology Department, in accordance with the United Kingdom Brain Bank criteria from 2016 to 2017. Patients with history of other neurologic or psychiatric illness, or psychotropic medication usage were excluded. The severity of PD was evaluated using the United Parkinson Disease Rating Scale (UPDRS) [16], and modified Hoehn and Yahr staging (H & Y) scale [17]. The UPDRS is evaluated via clinical observation and interview for multiple aspects of PD, such as mental dysfunction and mood (Part I), motor disability (Part II), and motor impairment (Part III). The modified H&Y scale (from stages 1 through 5, more severe in later stages) provides an evaluation of functional disability. In addition, 15 healthy volunteers (4 men and 11 women; mean age: 62.60 ± 4.85 years) with similar levels of education but no medical history of brain trauma, substance abuse, neurological diseases, or psychiatric illnesses were enrolled for comparison as normal controls.

### 2.2. Image Acquisition

#### 2.2.1. Thigh MRI Data Acquisition

We performed magnetic resonance scanning on a 1.5T MRI system (Discovery 450, GE Healthcare, Chicago, IL, USA). For signal excitation, a body coil was used; for signal reception, we used a 12-channel body phased array coil.

We employed a multi-echo 3D SPGR IDEAL sequence with fly-back gradients (IDEAL IQ, GE Healthcare) to evaluate the percentage of fat content in the thigh. The IDEAL IQ technique, a T1-independent, T2*-corrected chemical shift-based method, is capable of separating fat and water with multi-peak fat spectral modeling. Imaging parameters for IDEAL IQ were echo time = 1.3, 3.3, 5.3, 7.3, 9.3, and 11.3 milliseconds; flip angle = 5; bandwidth = 61.25 kHz; repetition time = 13.7 milliseconds; field of view = 42 × 42 cm at thigh region; matrix size = 256 × 128; slice thickness = 10 mm; and number of slices = 36 at thigh region. The IDEAL IQ produces water, fat, out-phase, in-phase, as well as T2*-corrected fat, T2*-corrected water, and fat fraction maps. IDEAL is a fat suppression sequence with the “in-and-out-of-phase” technique, which is based on Dixon’s method. The “in-and-out-of-phase” technique is acquired with different echo times (TE). Clear separation of these tissues is achieved by differences in chemical shift between water and fat. With the reduction of inhomogeneity of the B0 field, this method is gradually modified and improved. To make obtaining the best fat saturation possible, the lower extremities protocol included IDEAL sequences, as these sequences distinguish water from fat tissue (Water Only sequences and Fat Only sequences), and provide adequate fat saturation [15].

The fat fraction in muscles is approximately equal to signal intensity in the IDEAL fat fraction map image. The fat fraction of the thigh was estimated by calculating the signal intensity from regions of interest (ROI) in an IDEAL fat fraction map image. To ensure the appropriate position of regions of interest (ROI), all measurements were performed by two radiologists (LWC and WYN). The measured level of thigh was at the 50% point of femur length. The measured regions of interest (ROI) area included the anterior compartment of thigh (sartorius muscle and quadriceps femoris muscle: vastus lateralis, rectus femoris, vastus medialis, and vastus intermedius), the medium compartment of the thigh (gracilis muscle and adductor brevis, longus, minimus, and magnus), and the posterior compartment of the thigh (semitendinosus; semimembranosus; hamstring biceps femoris).

#### 2.2.2. Brain MRI Data Acquisition

A GE Signa 3T whole-body MRI scanner (General Electric Healthcare) using an 8-channel phase array head coil was used to perform the volumetric structural MRI scans. With 110 contiguous axial slices aligned to the anterior and posterior commissure, whole-brain 3-dimensional T1-weighted images of all participants were collected using an axial inversion-recovery prepared fast-spoiled gradient-recalled echo pulse sequence. The scanning parameters were as follows, repetition time = 9.5 m, echo time = 3.9 m; inversion time = 450 m, flip angle = 15°; number of excitations = 1; field of view = 240 × 240 mm^2^; matrix size = 512 × 512; and voxel size = 0.47 × 0.47 × 1.3 mm^3^ (without inter-slice gap and interpolation).

Voxel-based morphometry analysis was performed using the Statistical Parametric Mapping software (SPM12 version 7219, Wellcome Institute of Neurology, University College London, London, UK), and Matlab R2010a (Mathworks, Natick, MA, USA). First, all images were carefully checked by an experienced radiologist to ensure that no scanner artifacts, motion problems, or gross anatomic abnormalities existed for each participant. The default settings were used unless otherwise specified. Whole brain T1-weighted images were bias-corrected and segmented into gray matter, white matter, and cerebrospinal fluid using the New Segment Toolbox of SPM12. Then, the gray matter images were rigid aligned to the tissue probability maps in the Montreal Neurological Institute (MNI) standard space and averaged to create the study-specific tissue template using the high dimensional Diffeomorphic Anatomical Registration Exponentiated Lie (DARTEL) algorithm. Subsequently, all native space gray matter images were registered to this study-specific template and further spatially normalized into standard MNI space (1.5 mm isotropic voxel). The resulting gray matter images were modulated by Jacobian determinant of the corresponding deformation field to correct for volume changes. Finally, the modulated gray matter images were smoothed using an isotropic Gaussian kernel of 8 mm full-width at half maximum.

### 2.3. Statistical Analysis

All statistical analyses were performed using the Statistical Package for Social Sciences (SPSS) software package (version 17, SPSS Inc. Chicago, IL, USA). Age data for the study groups was compared using the independent *t*-test. Gender data for the study groups was compared using the Pearson Chi-square test. Analysis of covariance (ANCOVA) was used to analyze difference in clinical severity, percentage of fat content of the thigh, and global brain volume after adjustment for age and sex. All data were reported as mean ± the standard deviation (SD). Statistical significance was set at *p* < 0.05.

To examine between-group differences in regional gray matter volume, a voxel-wise general linear model was used to compare gray matter volume between PD group and normal control group using 1-factor 2-level ANCOVA design with age, sex, and total intracranial volume as covariates. The statistic threshold was set at cluster-level family-wise error (FWE) corrected *p*-value < 0.001, with a cluster size of at least 187 voxels, based on the results of a Monte Carlo simulation using the command-line tool of Analysis of Functional Neuro Images software (AFNI; Version AFNI_17.1.04; http://afni.nimh.nih.gov/afni/; 3d Cluster Sim with the following parameters: voxel *p*-value < 0.005, with explicit gray matter mask and 10,000 simulations). The regional gray matter volume of clusters with significant between-group differences were extracted and averaged for further correlation analysis.

Partial correlation analysis was performed with age and gender adjustments to determine the associations between the percentage of thigh fat content and regional gray matter volume. Furthermore, linear regression analysis adjusted for age and gender was performed for a more complete modeling of the relationship between the percentage of thigh fat content and gray matter volume changes.

## 3. Results

### 3.1. Demographic and Clinical Characteristics

The demographic and clinical data of the participants are shown in Table 1. The PD and normal control groups had similar mean age and gender distribution (age: *p* = 0.409; gender: *p* = 0.649). The mean UPDRS total score, modified H & Y, and disease duration (years) were 29.03, 1.30, and 1.13, respectively, for patients with PD.

### 3.2. Group Differences in Thigh Fat Percentages

The thigh fat percentages of the study participants are listed in Table 1. Compared with the normal control group, the PD group exhibited significantly elevated thigh fat percentage (mean ± SD: 12.02 ± 4.18 vs. 10.14 ± 2.40; *p* < 0.001).

### 3.3. Group Differences in Regional Gray Matter Volume Loss

**Figure 1:**
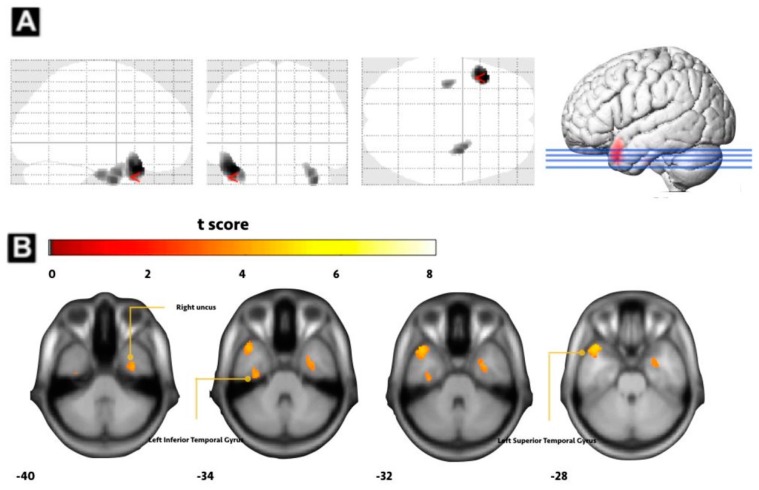
Voxel-based morphometry analysis between PD group and NC group. (**A**) Smaller gray matter volume (GMV) in PD group versus normal control (NC) group in the right uncus. (**B**) Left inferior temporal gyrus and left superior temporal gyrus (FWE corrected *p*-value < 0.001).

As shown in Figure 1, the PD group had significantly smaller total intracranial volume, grey and white matter volume than the normal control group. Furthermore, the PD group had a lower gray matter volume in several brain locations of the default mode network (DMN), including the left superior temporal gyrus, right uncus, and left inferior temporal gyrus compared to the normal control group (Figure 1 and Table 2).

### 3.4. Examination of the Relationship between Thigh Fat Percentage and Regional Gray Matter Volume

**Figure 2:**
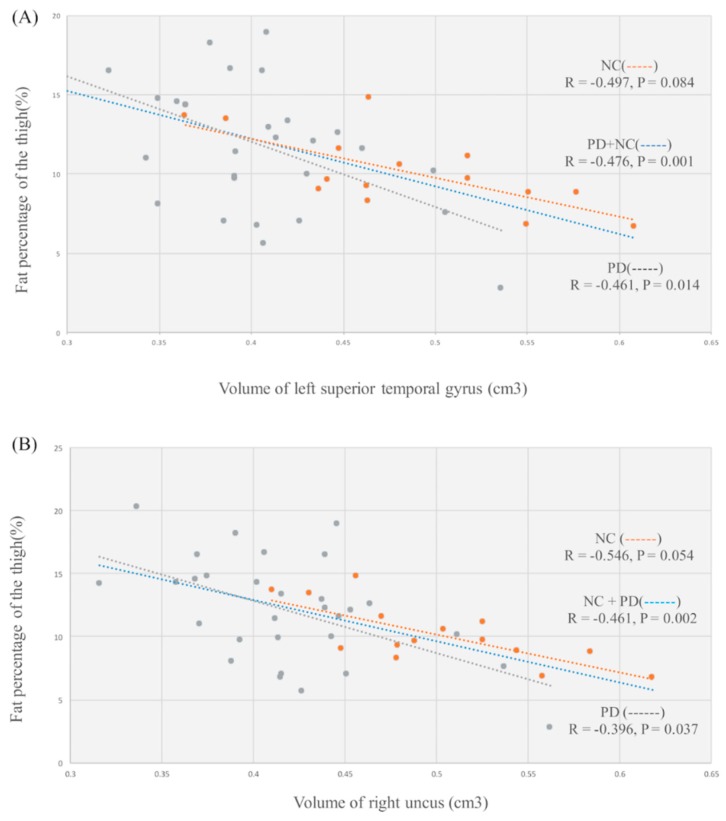
Relationship between thigh fat percentage and regional gray matter volume (GMV). (**A**) Lower gray matter volume of the left superior temporal gyrus was associated with increase in fat percentage of thigh. (**B**) Lower gray matter volume of the right uncus was associated with increase in fat percentage of thigh.

The increased thigh fat percentage was associated with lower gray matter volume of the left superior temporal gyrus (*p* = 0.001, *r* = −0.476), and right uncus (*p* = 0.002, *r* = −0.461) (shown by blue line in Figure 2). Furthermore, in patients with PD, increased thigh fat percentage was associated with lower gray matter volume of the left superior temporal gyrus (*p* = 0.014, *r* = −0.461), and right uncus (*p* = 0.037, *r* = −0.396) (shown by gray line in Figure 2). There was no significant correlation between thigh fat percentage and regional gray matter volume in healthy participants (shown by orange line in Figure 2). Furthermore, we performed regression analyses to investigate whether regional gray matter volume loss (left superior temporal gyrus, right uncus) or clinical variables such as age at disease onset, gender, and disease duration influenced the detected fat percentage of the thigh. The results revealed that the female gender (β = −0.304, *p* = 0.012) and disease duration (β = 0.397, *p* = 0.002) were associated with thigh fat percentage. Of note, the regression analyses revealed no significant correlation between thigh fat percentage and volume of left inferior temporal gyrus.

## 4. Discussion

### 4.1. Summary

Consistent with previous studies and in confirmation of our hypothesis, we found that compared with healthy controls, PD patients have increased fat infiltration of core muscle, globally decreased gray matter, white matter, and intracranial volume, particularly in the left superior temporal gyrus, right uncus, and left inferior temporal gyrus. Furthermore, the study also revealed correlations between fat infiltration of core muscle, which represents core muscle loss, and grey matter atrophy. The results of this study indicate grey matter atrophy in the superior temporal gyrus and uncus, are associated with core muscle loss. Finally, linear regression study revealed small superior temporal gyrus volume, female gender, and longer disease duration are associated with core muscle loss in patients with PD.

### 4.2. Muscle Atrophy of PD Patients May Not Solely Result from PD-Related Movement Symptoms

Using the MRI chemical shift analysis in the present study, we found that PD patients presented elevated fatty infiltrates in the bilateral thigh as compared to healthy controls. This finding is consistent with a previous study which reported that PD patients have a higher prevalence rate of physical frailty than the normal aging population [18]. Notably, the fat infiltration in PD patients was not presented at the lesion side only. The non-lesion side of the thigh also showed a higher fat percentage; in fact, no significant differences were observed between the lesion side and the non-lesion side within the PD group. This indicates the muscle loss may not directly come from the PD-related movement symptoms only; there may be other pathophysiology shared between PD and the muscular system affecting the muscle loss in patients with PD. Factors such as lifestyle alteration caused by decreasing motor function [8], malnutrition caused by movement and swallowing problems [19], and neuroinflammation [20] may also be involved. To be clear, the link between PD and core muscle loss may initially be driven by neuroinflammation. According to a previous study, elevated levels of inflammatory mediators have been detected in both early-stage PD and sarcopenia, [19]. Moreover, alpha-synuclein deposition, the histologic hallmark of PD, is evident not only in the central nervous system but also involves structures belonging to the peripheral nervous system [21]. Finally, the deterioration of the associated brain networks, such as the executive attention network [9] or default mode network [10,11] may lead to movement problems in PD patients, thereby further lessening physical activity and causing a higher risk of core muscle loss.

### 4.3. Effects of Clinical Variables on Core Muscle Fatty Infiltration in PD Patients

To verify the cause and predictive factor of fatty infiltration in PD patients, we used two statistical methods: partial correlation analysis and linear regression analysis. Prior to those, VBM was used to measure the total and regional brain volume difference between PD patients and healthy volunteers. The results indicate that PD patients had globally decreased gray matter volume, especially in left superior temporal gyrus, right uncus, and left inferior temporal gyrus compared to healthy controls. This is indeed consistent with previous studies reporting that physical frailty and sarcopenia are linked to brain structure changes [22,23], suggesting the role of the central nervous system in the pathophysiology of physical frailty [12]. Partial correlation analysis revealed the gray matter volume reductions in specific regions, such as the uncus and superior temporal gyrus, were significantly associated with fatty infiltration in PD patients. Further linear regression analysis revealed that aside from volume reduction in those specific regions, female gender and longer disease duration were factors predictive of core muscle fatty infiltration in patients with PD. Of note, disease progression may affect both muscle loss and brain damage. Although this study revealed correlations between fat infiltration of core muscle and grey matter atrophy, the specific cause and effect relationship remains unclear.

Female gender as a risk factor of core muscle loss may be influenced by a multifactorial process, including hormonal alterations and nutrition [24]. Androgen plays an important role in the maintenance of muscle mass, whereas low plasma testosterone levels can cause or accelerate muscle- and age-related diseases, such as sarcopenia [25]. Previous studies have shown that the healthy female population generally has higher fat mass percentage, extremity fat and subcutaneous fat, with lower lean mass and skeletal muscle mass compared to men; meanwhile, males have higher visceral adipose tissue and intramyocellular lipids than women [26,27,28,29]. Given these gender body composition differences, it is problematic to determine whether the association of fat percentage with female gender identified in this study is normal or due to disease pathology. Therefore, gender should be considered a control factor in future studies. Furthermore, in this study there were only 10 male patients in the PD group and four male normal controls, potentially biasing the results due to imbalanced sample size.

Longer disease duration is also a risk factor of core muscle loss. As the disease progresses, PD patients may develop several comorbidities, such as gastrointestinal dysfunction, dysphagia [19], gastroparesis [30], constipation [31], cognitive impairment, and depression [32], which cause inadequate energy input in PD patients. In contrast, tremors, rigidity, and dyskinesia increase energy output [33]. Decreasing energy input and increasing energy output results in weight loss and muscle mass reduction in PD patients over time [34].

### 4.4. Possible Effects of Brain Structural Changes on Core Muscle Fatty Infiltration in PD

Volume reductions in several brain regions, such as the uncus and superior temporal gyrus are a risk factor of core muscle loss in PD patients. Although studies have reported that these brain regions are associated with decreased muscle mass, weakness, muscle dysfunction, and sarcopenia [12,22,23], the precise associations between these brain regions, and the mechanisms causing core muscle loss in PD patients, have yet to be elucidated.

The performance of motor functions depends on the synergetic interaction between different networks of the brain, among which the default mode network plays a critical role [35]. The default mode network is comprised of the medial prefrontal cortex, inferior parietal cortex, precuneus, posterior cingulate cortex [36,37], and the medial temporal lobe [38,39]. In this study, fat infiltration in PD patients was associated with decreased volumes of the superior temporal gyrus and uncus, which are also constituent parts of the default mode network [40]. It has been reported that the default mode network region has higher blood flow than other brain regions in the resting state [41]; however, during task performance, the cerebral blood flow in the default mode network region is reduced, while the blood flow in other task-related networks is increased [42]. As such, the default mode network acts as a “functional preserver” in the brain. It has been suggested that brain volume loss is associated with decreased cerebral blood flow [43]. In the healthy brain, the task-related network increases activation in response to external stimuli [44], while default mode network activity is suppressed. However, in PD patients the volume of default mode network is reduced [45,46], equating to a decreased role as a “functional preserver” [43], causing the task-related network to not effectively activate when performing a task, consequently resulting in poor motor function. This may be one of the reasons for core muscle loss in PD patients. However, more evidence is required to clarify the network interactions within the brain of PD patients.

### 4.5. Limitations

Although this study achieved some meaningful findings, limitations indeed exist. First, although MRI scans can precisely estimate lean mass change, it is difficult to apply to a large sample of subjects, and as participants were recruited from a single center, they may not be representative of the PD population as a whole. Second, while there were 30 PD patients, only 15 normal controls were enrolled in our study; as such, caution regarding interpretation of the results is warranted due to imbalanced sample size. Finally, future longitudinal studies may be necessary to further clarify the causal relationship between gray matter volume loss and sarcopenia.

## 5. Conclusions

Core muscle loss is common in PD patients, the identification of which may lead to morbidity and mortality, potentially altering individual patient treatment plans. The present study reveals an association between core muscle loss and brain structural changes to the superior temporal cortex, uncus cortex, and inferior temporal cortex, indicating that the pathophysiology of PD and core muscle loss may be consequentially related processes involving specific pathways and factors. These may act as biomarkers for the early identification and prevention of sarcopenia in PD patients.

## Figures and Tables

**Table 1:** Demographic and clinical data and fat percentage of core muscle of PD group and normal control group.

Demographics	PD (*n* = 30)	NC (*n* = 15)	*p*-Value
Age (years)	64.90 ± 10.08	62.60 ± 4.85	0.409
Gender (F/M)	20/10	11/4	0.649
GMV	564.3 ± 63.0	603.3 ± 50.5	<0.001
WMV	419.7 ± 64.3	422.7 ± 40.4	<0.001
TIV	1314.3 ± 129.6	1350.7 ± 135.9	<0.001
**Disease Severity Scale**			
Disease duration	1.13 ± 1.36		
HY stage	1.30 ± 0.45		
UPDRS-I	2.90 ± 2.12		
UPDRS-II	7.00 ± 4.30		
UPDRS-III	19.13 ± 11.73		
UPDRS-176	29.03 ± 16.10		
**Fat Percentage of Thigh (%)**			
Non-lesion side	11.80 ± 4.15	10.14 ± 2.40 *	<0.001
Lesion side	12.23 ± 4.38	10.14 ± 2.40 *	<0.001
Left side	12.10 ± 3.98	10.25 ± 2.47	<0.001
Right side	11.30 ± 4.04	10.03 ± 2.38	<0.001
Total	12.02 ± 4.18	10.14 ± 2.40	<0.001

Gender data were compared by Pearson Chi square test. Age and fat percentage of core muscle were compared by independent *t*-test after controlling for age and gender. Data are presented as mean ± standard deviation. * The values of non-lesion side and lesion side of fat percentage in normal control (NC) group were calculated with the average on both side. PD = Parkinson’s disease, GMV = gray matter volume, WMV = white matter volume, TIV = total intracranial volume, UPDRS = United Parkinson’s Disease Rating Scale, Modified H & Y = Modified Hoehn and Yahr stages (maximum score is 5).

**Table 2:** Regions of statistically significant reduced gray matter volume (GMV) in early PD compared to healthy control group.

Region	MNI Coordinates	Voxel Size	GMV, mm^3^		*t*-Value
	X, Y, Z		PD Group	NC Group	
Superior temporal gyrus, L	−42, 18, −28	890	399.64 ± 55.33	484.67 ± 68.41	4.83
Uncus, R	34, −4, −40	461	420.08 ± 53.75	501.25 ± 57.93	4.27
Inferior temporal gyrus, L	−34, −16, −34	188	446.43 ± 64.39	525.71 ± 49.64	4.07

GMV = grey matter volume, PD = Parkinson’s disease, L = left, R = right, MNI = Montreal Neurological Institute. The statistical criteria were set at joint voxel height uncorrected *p* < 0.001 and a cluster extent threshold of the family-wise error rate (PFWE) < 0.05.
